# The Essential Role of PRAK in Preserving Cardiac Function and Insulin Resistance in High-Fat Diet-Induced Diabetes

**DOI:** 10.3390/ijms22157995

**Published:** 2021-07-27

**Authors:** Jianfeng Du, Yu Tina Zhao, Hao Wang, Ling X. Zhang, Gangjian Qin, Shougang Zhuang, Marshall Kadin, Y. Eugene Chin, Paul Y. Liu, Ting C. Zhao

**Affiliations:** 1 Department of Surgery, Roger Williams Medical Center, Boston University School of Medicine, Providence, RI 02908, USA; email2du@gmail.com (J.D.); wanghaolxf@hotmail.com (H.W.); lzhang1@lifespan.org (L.X.Z.); 2 University of Rochester School of Medicine and Dentistry, Rochester, NY 14642, USA; tinazhao24@gmail.com; 3 Department of Biomedical Engineering, University of Alabama at Birmingham, Birmingham, AL 35294, USA; gqin@uab.edu; 4 Department of Medicine, Rhode Island Hospital, Alpert Brown Medical School, Brown University, Providence, RI 02903, USA; SZhuang@lifespan.org; 5 Department of Plastic Surgery, Rhode Island Hospital, Brown University, Providence, RI 02903, USA; mkadin@me.com (M.K.); pliu@lifespan.org (P.Y.L.); 6 Department of Pathology, Rhode Island Hospital, Alpert Brown Medical School, Brown University, Providence, RI 02903, USA; 7 Institute of Health Sciences, Chinese Academy of Sciences-Jiaotong University School of Medicine, Shanghai 200031, China; yechin@sibs.ac.cn

**Keywords:** PRAK, insulin resistance, myocardium, high-fat diet, metabolic stress

## Abstract

Regulated/activated protein kinase (PRAK) plays a crucial role in modulating biological function. However, the role of PRAK in mediating cardiac dysfunction and metabolic disorders remains unclear. We examined the effects of deletion of PRAK on modulating cardiac function and insulin resistance in mice exposed to a high-fat diet (HFD). Wild-type and PRAK^−/−^ mice at 8 weeks old were exposed to either chow food or HFD for a consecutive 16 weeks. Glucose tolerance tests and insulin tolerance tests were employed to assess insulin resistance. Echocardiography was employed to assess myocardial function. Western blot was used to determine the molecular signaling involved in phosphorylation of IRS-1, AMPKα, ERK-44/42, and irisin. Real time-PCR was used to assess the hypertrophic genes of the myocardium. Histological analysis was employed to assess the hypertrophic response, interstitial myocardial fibrosis, and apoptosis in the heart. Western blot was employed to determine cellular signaling pathway. HFD-induced metabolic stress is indicated by glucose intolerance and insulin intolerance. PRAK knockout aggravated insulin resistance, as indicated by glucose intolerance and insulin intolerance testing as compared with wild-type littermates. As compared with wild-type mice, hyperglycemia and hypercholesterolemia were manifested in PRAK-knockout mice following high-fat diet intervention. High-fat diet intervention displayed a decline in fractional shortening and ejection fraction. However, deletion of PRAK exacerbated the decline in cardiac function as compared with wild-type mice following HFD treatment. In addition, PRAK knockout mice enhanced the expression of myocardial hypertrophic genes including ANP, BNP, and βMHC in HFD treatment, which was also associated with an increase in cardiomyocyte size and interstitial fibrosis. Western blot indicated that deletion of PRAK induces decreases in phosphorylation of IRS-1, AMPKα, and ERK44/42 as compared with wild-type controls. Our finding indicates that deletion of PRAK promoted myocardial dysfunction, cardiac remodeling, and metabolic disorders in response to HFD.

## 1. Introduction

The mitogen-activated protein (MAP) kinase pathway has been identified to contribute critically to the regulation of metabolic stress and tissue injury in multiple tissues [1]. Four distinct conventional MAPK pathways have been classified in mammals, which include p38 MAPK, extracellular signal-regulated kinases (ERKs) 1 and 2 (ERK1/2), c-Jun *N*-terminal kinases 1/2/3 (JNK1/2/3), and ERK5 [2,3]. We demonstrated that activation of p38 and MAPKAP kinase-2 induced a preconditioning effect in response to myocardial ischemia and reperfusion injury [4,5,6]. There is evidence showing that metabolic stresses manifested an increase in the phosphorylation of p38, which is related to the insulin signaling pathway [7,8,9]. p38 regulated/activated kinase (PRAK) is identified as a direct substrate of p38 MAPK, although it can be activated by atypical MAPKs and ERK3/4 [9,10,11,12,13]. Relative to investigating the role of p38, the function of PRAK has not been fully investigated for its modulation of metabolism and insulin resistance. We recently demonstrated that ablation of PRAK increased susceptibility of the myocardium against myocardial ischemia and reperfusion injury and increased myocardial dysfunction and enhanced myocardial remodeling in chronic myocardial infarction. Interestingly, there is evidence that a specific p38 kinase cascade, p38β-PRAK, is essential for energy depletion-induced inactivation of mammalian target of rapamycin complex 1 (mTORC1) [14]. In addition, depletion of PRAK in mice promotes 7,12-dimethylbenz[a]-anthracene (DMBA)-induced skin carcinogenesis, coinciding with development of compromised senescence [15]. However, it remains unknown if PRAK is essential to modulating myocardial function and metabolic stress in response to high-fat diet-induced diabetes. In this study, we sought to determine if deletion of PRAK could promote myocardial dysfunction, cardiac remodeling, and insulin resistance following HFD intervention. We also sought to determine if PRAK deletion could induce an impairment in the insulin signaling pathway. Our results indicate that deletion of PRAK promoted myocardial dysfunction, enhanced myocardial remodeling, suppressed the insulin signaling pathway, and elicited insulin resistance in response to HFD, suggesting that PRAK is critical to modulating cardiac function and remodeling in mice exposed to HFD.

## 2. Results

### 2.1. PRAK Knockout-Enhanced HFD Induces Glucose Intolerance

**Figure 1:**
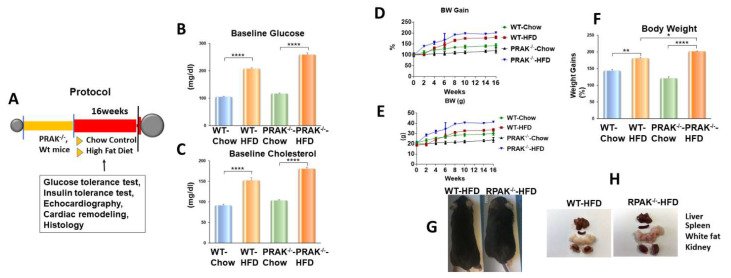
PRAK deletion enhances diet-induced metabolic syndrome in obese mice. (**A**) Schemas of animal experimentation in high-fat diet (HFD) versus chow control diet in both wild-type (Wt) and PRAK^−/−^ mice; (**B**,**C**) Total blood cholesterol and glucose concentration of mice fed with standard chow or HFD after 16 weeks; (**D**–**F**) Body weight gain of mice exposed to chow food and a high fat diet in wild type and PRAK^−/−^ mice; (**G**,**H**) Mouse and organs. Data are shown as means ± SEM * *p*< 0.05, ** *p* < 0.01; **** *p* < 0.0001 (*n* = 5/each group).

**Figure 2:**
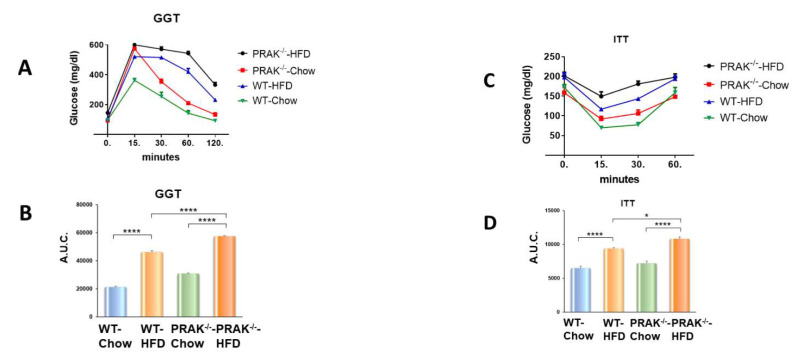
Deletion of PRAK enhances insulin resistance in mice exposed to HFD. (**A**,**B**) Glucose tolerance test. Area under curve for GTT. Data are shown as means ± SEM; **** *p* < 0.0001 (n = 5/each group). (**C**,**D**) Insulin tolerance test (ITT). Data are shown as means ± SEM * *p* < 0.05, **** *p* < 0.0001 vs. HFD. Area under curve for ITT. **** *p* < 0.0001 (*n* = 5/ each group).

As shown in Figure 1A, PRAK^wt^ or PRAK^−/−^ mice were exposed to both chow and high-fat diet for 16 weeks to induce metabolic stress. As shown in Figure 1B,C, PRAK^−/−^ mice displayed more severe hyperglycemia and hypercholesterolemia as compared with wild-type mice exposed to a high-fat diet (HFD), as indicated by the increases in blood glucose and cholesterol levels following dietary interventions. Moreover, as shown in Figure 1D–F, there was no significant difference in body weight prior to food interventions. However, as expected, mice developed obesity upon exposure to HFD as compared with chow control food. However, deletion of PRAK resulted in a much greater significant increase in body weight as compared with wild-type HFD mice (Figure 1G), which is consistent with the larger organs (Figure 1H). In order to determine the effect of deletion of PRAK on insulin resistance, we implemented glucose tolerance tests and insulin tolerance tests. As show in Figure 2A, wild-type and PRAK^−/−^ mice fed with standard chow food demonstrated a normal glucose response as evident by measurements of the glucose tolerance test. However, wild-type mice displayed glucose intolerance, as indicated by GTT as well as by the calculated area under the curve (AUC) (Figure 2A,B) when exposed to HFD. Deletion of PRAK exacerbated glucose intolerance as indicated by GTT and AUC when exposed to HFD. In addition, as shown in Figure 2C,D, there is normal insulin tolerance in both wild-type and PRAK^−/−^ mice exposed to control chow food. However, deletion of PRAK exacerbated the magnitude of insulin intolerance, which is indicated by ITT and AUC that were enhanced by depletion of PRAK in response to HFD.

### 2.2. PRAK Knockout-Enhanced HFD Induces Left Ventricular Dysfunction

**Figure 3:**
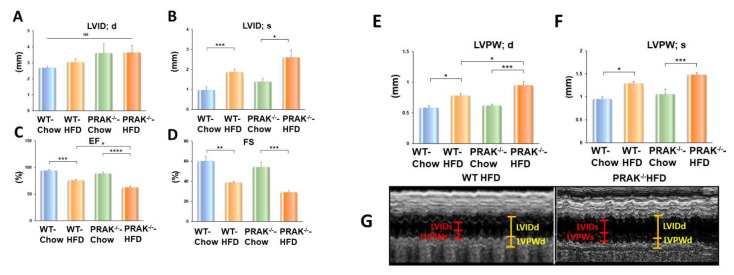
PRAK deletion exacerbates impairment in left ventricular function in HFD-fed mice. Echocardiographic measurements of ventricular functional parameters include (**A**,**B**) Left Ventricular Internal Dimension in Diastole (LVID;d); Left Ventricular Internal Dimension in Systole (LVID;s); Data are shown as means ± SEM (*n* = 5/each group). * *p* < 0.05, *** *p* < 0.001; (**C**,**D**): Ejection Fraction (EF), Fractional Shortening (FS); Data are shown as means ± SEM (*n* = 5/each group). * *p* < 0.05, ** *p* < 0.01, *** *p* < 0.001, **** *p* < 0.0001. (**E**,**F**): Left ventricular posterior wall (LVPW;d and LVPW;s; Data are shown as means ± SEM (*n* = 5/each group). * *p* < 0.05, *** *p* < 0.001. (**G**): M-Mode showing the promotion of ventricular enlargement in PRAK^−/−^ mice exposed to HFD as compared to wild type receiving HFD group.

**Figure 4:**
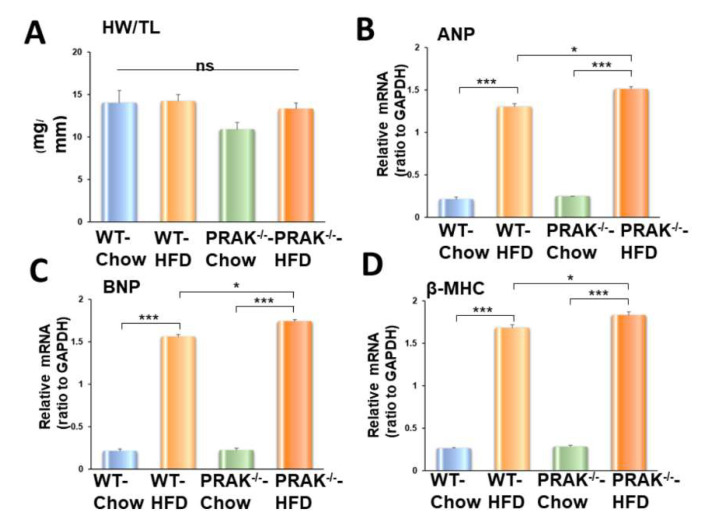
Depletion of PRAK enhances hypertrophic gene expression in mice exposed to HFD. (**A**): Heart weight/tibia length ratio. Data are shown as means ± SEM (*n* = 5/each group). (**B**): *Atrial natriuretic peptide* (ANP); (**C**): *B-type natriuretic peptide* (BNP); (**D**): *Myosin heavy chain* beta (β-MHC); Data are shown as means ± SEM (*n* = 3–4/each group). * *p* < 0.05 *** *p* < 0.001.

Echocardiographic parameters were used to assess ventricular function in the tested animals. There was no significant difference in myocardial function among the groups prior to dietary intervention. Mice fed with HFD exhibited an increase in left ventricular internal dimension (LVID) in both the systolic and diastolic stages as compared with the LVID of mice exposed to the chow-food control group (Figure 3A,B). Furthermore, as shown in Figure 3C,D, wild-type mice fed with HFD demonstrated myocardial dysfunction, which is indicated by a significant decrease in ejection fraction and fractional shortening as compared with the chow control group (* *p* < 0.05). However, depletion of PRAK exacerbated the magnitude of cardiac dysfunction in mice exposed to HFD. Likewise, wall thickness (LVPW) was augmented in HFD-fed animals, which was exacerbated by the deletion of PRAK (Figure 3E,F). PRAK deletion-induced ventricular enlargement is also shown in Figure 3G. PRAK^−/−^ mice showed an increase in heart weight/tibia length ratio in response to the HFD condition as compared with wild-type mice (Figure 4A). Furthermore, fetal genes including ANP, BNP, and βMHC were induced by exposure to HFD, while deletion of PRAK aggravated expression of these hypertrophic genes in the heart (Figure 4B–D).

### 2.3. PRAK Deletion Increased Interstitial Fibrosis and Enhanced Myocyte Hypertrophy in the Myocardium

**Figure 5:**
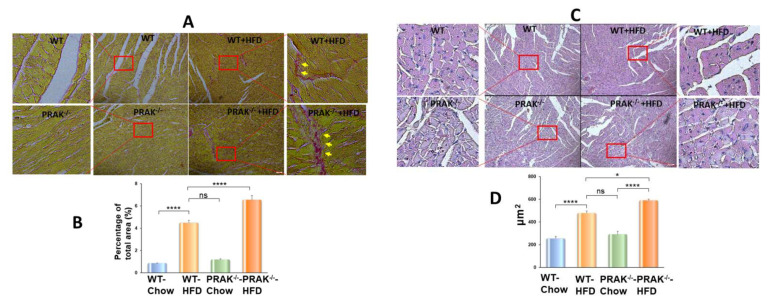
PRAK deletion increases interstitial fibrosis and enhances myocyte hypertrophy in the myocardium. (**A**,**B**): Interstitial fibrosis was evaluated by picrosirius red staining in hearts from wild-type (WT) and PRAK^−/−^ mice in response to chow and HFD (*n* = 3–4/per group), respectively. Data represent means  ±  SE (*n* =3–4/per group), **** *p* < 0.0001. Representative images of picrosirius red staining in hearts. (**B**): Scale bar  =  50 μm). (**C**,**D**)**:** Assessment of myocyte size of myocardium in each group, respectively. Data represent means  ±  SE (*n* = 3–4/ per group). * *p* < 0.05, **** *p* < 0.0001. Representative images of H&E staining in hearts (Scale bar  =  50 μm).

**Figure 6:**
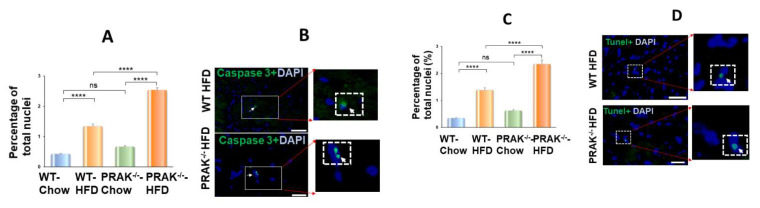
PRAK deletion increases apoptotic frequency in the myocardium. (**A**,**B**) Extent of apoptosis in hearts were evaluated by staining with cleaved caspase 3 (*n* = 3–4/per group).Data represent means  ±  SE. **** *p* < 0.0001. (**C**,**D**) Extent of apoptosis in hearts were evaluated by terminal deoxynucleotidyl transferase nick-end labeling (TUNEL) assay (*n* = 3–4/per group). **** *p* < 0.0001.

Picrosirius red was used to estimate interstitial fibrosis in the myocardium. There was no significant picrosirius red staining in either wild-type or PRAK^−/−^ mice exposed to chow food. However, wild-type mice fed with HFD showed an increase in interstitial fibrosis in the heart (Figure 5). HFD also augmented interstitial fibrosis in the heart in PRAK^−/−^ mice (Figure 5A,B). The density measurements indicate that deletion of PRAK caused a significant increase in interstitial fibrosis in response to HFD, which was significantly higher than that of HFD-fed wild-type mice (* *p* < 0.0001 vs. WT-HFD). As shown in Figure 5C,D, HFD induced an increase in myocyte size as compared with chow control in both wild-type and PRAK^−/−^ groups, but deletion of PRAK induced a marked increase in interstitial fibrosis as compared with the wild-type HFD group (*p* < 0.05 vs. WT-HFD). As shown in Figure 6A,B, there is no detectable active caspase-3 positive signal in the myocardium obtained from either the wild-type or PRAK^−/−^ chow group, but PRAK^−/−^ HFD resulted in a statistical increase in active caspase-3 positive signals as compared with wild-type HFD (*p* < 0.001 vs. WT-HFD). Likewise, as shown in Figure 6C,D, deletion of PRAK also resulted in an increase in TUNEL positive signals in the hearts (*p* < 0.001 vs. WT- HFD).

### 2.4. PRAK Deletion Resulted in a Decrease in Phosphorylation of IRS-1, AMPKα, and ERK1/2 and in a Reduction of Irisin

**Figure 7:**
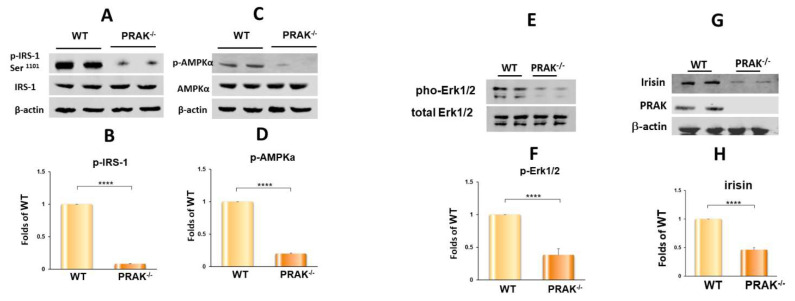
PRAK deletion decreases phosphorylation of IRS-1, AMPKα, and ERK1/2 in the myocardium. (**A**,**B**) Representative immunoblot of phosphorylated IRS-1 and total IRS-1 and densitometric analysis; (**C**,**D**) Representative immunoblot of phosphorylated AMPKα and total AMPKα and densitometric analysis; (**E**,**F**) Representative immunoblot of phosphorylated ERK1/2 and total ERK1/2 and densitometric analysis; (**G**,**H**) Representative immunoblot of irisin, PRAK and densitometric analysis of irisin; Data represent means  ±  SE (*n* = 4/ per group). **** *p* < 0.0001.

As shown in Figure 7A,B, the myocardia in wild-type mice show phosphorylation of IRS-1; deletion of PRAK decreased the phosphorylation of IRS-1 (*p* < 0.01 vs. WT), but there was no reduction in total IRS-1. Furthermore, as shown in Figure 7, phosphorylation of both AMPKα (Figure 7C,D) (*p* < 0.01 vs. WT) and ERK1/2 (Figure 7E,F) (*p* < 0.01 vs. WT) in the myocardia was also suppressed by deletion of PRAK, while there is no difference in total AMPKα and ERK1/2 levels. In addition, deletion of PRAK confirmed the absence of PRAK protein in myocardia (Figure 7G). As compared with the wild-type group, deletion of PRAK resulted in the reduction of irisin, a myokine in the myocardium (Figure 7G,H).

## 3. Discussion

Salient findings: This is the first demonstration to show that PRAK plays a critical role in developing cardiac dysfunction and metabolic disorders in mice exposed to a high-fat diet. Using PRAK knockout and wild-type mouse models, we show that deletion of PRAK promoted myocardial ventricular dysfunction in mice exposed to HFD, which was associated with the enhancement of cardiac hypertrophy, increased cardiac hypertrophic genes, and the augmentation of myocardial remodeling. Furthermore, depletion of PRAK elevated insulin resistance and exacerbated hyperglycemia and hypercholesterolemia in HFD-fed mice. Depletion of PRAK promoted interstitial fibrosis in the myocardium and increased apoptotic signaling in the myocardium as compared with the wild-type group. Furthermore, phosphorylation of IRS-1, AMPKα, and ERK1/2 were significantly suppressed in the myocardium following deletion of PRAK, which was also consistent with the decrease in irisin, a myokine for the regulation of muscle metabolism. Our results indicate that PRAK plays a critical role in mediating myocardial function in high-fat diet-induced metabolic stress.

It was reported that a specific p38 kinase cascade/PRAK plays a critical role for energy depletion-induced inhibition of mTORC1 [14,16,17,18]. The phosphorylation of the mTORC1 regulatory-associated protein is closely related with the modulation of AMPK [19,20,21,22]. In an agreement with this report, our study revealed that deletion of PRAK suppressed AMPK phosphorylation in the myocardium, indicating the regulatory effect of PRAK on AMPK activation. In addition, we found that deletion of PRAK resulted in decreases in the phosphorylation of IRS-1, a key component in the insulin signaling pathway as compared with wild-type control, suggesting the direct relationship of PRAK and IRS-1 in mediating insulin signaling. Although a relationship between p38 MAPK and ERK1/2 has been extensively studied in a variety of models [23], there is little information on whether PRAK also mediates ERK1/2 phosphorylation in regulating cardiac function in diabetes. We found that ERK1/2 phosphorylation was attenuated by knockout of PRAK, indicating the correlation between PRAK and ERK1/2 in the myocardium.

Obesity and insulin resistance was in part mediated by the stimulation of MAPKs, including p38 MAPK or induction of the expression of MAPK phosphatase-1 (MKP-1). Mice lacking MKP-1 (MKP1-MKO) in skeletal muscle demonstrated increased skeletal muscle p38 MAPK, which manifested in resistance to the development of obesity in mice exposed to dietary interventions [24]. Another observation shows that mice lacking p38γ/δ in myeloid cells displayed resistance to the development of diet-induced fatty liver and glucose intolerance, which contributes to p38 deficiency in neutrophil infiltration, thereby promoting the development of steatosis and liver metabolic changes [25].

We recently found that knockout of PRAK enhanced myocardial ischemia and reperfusion injury and promoted myocardial remodeling, which is associated with the reduction of ERK1/2 phosphorylation in the myocardium [26]. In agreement with the observation about the role of PRAK in myocardial ischemia, PRAK deletion exacerbated myocardial dysfunction and promoted myocardial remodeling under the condition of HFD, indicating the critical role of PRAK in preservation of cardiac performance and that PRAK might share a similar functional role in myocardial infarction and high-fat diet intervention. PRAK lays downstream of p38 MAPK, which plays different roles in regulating myocardial injury and the diabetic heart [27,28]. However, it still remains unknown if PRAK and p38 could function differently in modulating cardiac performance in cardiac injury and the diabetic heart. Our observation provides important evidence showing that PRAK is required to preserve cardiac performance in HFD-induced diabetes. The mechanism of deletion of PRAK in attenuating myocardial function is not clear, but reduction of irisin by the deletion of PRAK could be one of the most important factors responsible for mediating cardiac function. We previously reported that irisin improved myocardial functional recovery following myocardial ischemia and reperfusion injury [26]. Additionally, p38 is upstream of PCG-1α, resulting in the stimulation of FNDC5. A cleavage product of the extracellular portion of FNDC5 is secreted and circulated into peripheral circulation to act as a myokine [29,30,31]. Activation of p38 during exercise resulted in greater phosphorylated and activated PCG-1α, leading to the expression of mitochondrial proteins [32]. p38 MAPK is identified to directly phosphorylate the repressor regions of PCG-1α that act directly upstream of irisin [33,34]. This is in agreement with studies establishing that p38 plays a key role in mediating the production of PCG-1 [35,36,37,38,39]. It is likely that reduction of irisin in the myocardium contributes to promoting cardiac dysfunction in high-fat diet-induced diabetes. There is little evidence to show that knockout of PRAK mediates insulin resistance and metabolic disorder in diabetes. In this study, we found that deletion of PRAK promoted metabolic stress by illustrating hyperglycemia and hyperlipidemia in mice exposed to HFD, suggesting the critical contribution of PRAK in modulating metabolism. We did not monitor the effect of the food consumption by deletion of PRAK following dietary intervention; this deficiency prevents us from estimating the function of PRAK in modulating energy metabolism. Likewise, measurements of metabolic rate such as oxygen consumption, carbon dioxide production, respiratory exchange ratio, energy expenditure, and heat production in a PRAK^−/−^ model following dietary intervention in the future will provide more interesting information. PRAK knockout augmented systemic insulin resistance in HFD-fed mice. The mechanism by which PRAK knockout induced insulin resistance is likely to be related to the down-regulation of irisin and the suppression of IRS-1, AMPK α, and ERK1/2 phosphorylation since the myocardium of PRAK knockout mice showed reductions in irisin and IRS-1, AMPKα, and ERK phosphorylations. These components are responsible for regulating insulin sensitivity [40]. HFD-fed mice exhibited cardiac dysfunction, which was related to the impairment in insulin sensitivity [41]. PRAK knockout exacerbated myocardial dysfunction, which also may be likely attributed to the attenuation in insulin signaling such as IRS-1, AMPK, and ERK1/2. The potential important aspects of this study provide useful information with clinical implications in which specific targets of PRAK could serve as effective therapies to treat diabetes, metabolic disorders, obesity, and cardiovascular disease.

## 4. Materials and Methods

### 4.1. Reagents and Antibodies and Animals

4,6-Diamidino-2-phenylindole (DAPI) was obtained from Life Technologies (Grand Island, NY, USA). Terminal deoxynucleotidyl transferase nick-end labeling (TUNEL) kits were purchased from Trevigen (Gaithersburg, MD, USA). The primers used in this study were synthesized from Sigma-Aldrich (St. Louis, MO, USA). Male PRAK^−/−^ and litter mate control mice (C57BL/6J background) at the age of 8 weeks were used in this study. PRAK^−/−^ mice were obtained from the Scripps Research Institute (La Jolla, CA, USA) and bred in house. The genetic knockout of PRAK on mice was described previously, in which a targeting vector was made to replace exon 8 of the PRAK gene [15]. All studies on animals were performed under a protocol approved by the Institutional Animal Care and Use Committee, which conforms to the Guide for the Care and Use of Laboratory Animals published by the US National Institutes of Health (NIH Publication No. 85–23, revised 1996); these animals are housed in an accredited facility at Roger Williams Medical Center (Providence, RI, USA). The protocol for chow and high-fat diet treatment was described in Section 4.2. All animal procedures were carried out in accordance with guidelines approved by the Institutional Animal Care and Use Committee of Roger Williams Medical Center.

### 4.2. Animal and Experimental Protocol

Adult wild-type and PRAK^−/−^ mice were used to perform the proposed studies. All mice were housed at the Animal Care Facility of Roger Williams Medical Center in a temperature-controlled room (22 °C) on a 12 h light–dark cycle. Both wild-type control and PRAK^–/–^ mice were then randomly divided into two groups and were fed with either a high-fat diet (60% fat/20% carbohydrate/20% protein, Research Diets, New Brunswick, NJ, USA) or standard chow food (10% fat/70% carbohydrate/20% protein, New Brunswick, NJ, USA) for 16 weeks. Mice were sacrificed at the end of the 16 weeks, and tissues were collected for subsequent experiments.

### 4.3. Metabolic Measurements

Glucose tolerance tests (GTT) and insulin tolerance tests (ITT) were administered for the determination of insulin resistance. Mice were fasted for 4 h, and tail vein blood was collected for measurement of baseline glucose level using an Accu-Chek Compact Plus glucometer (Roche Diagnostics, Indianapolis, IN, USA). Mice then received a subsequent injection of glucose at a concentration of 1 mg/kg intraperitoneally. Glucose levels in the blood were assessed at 15, 30, 60, 120, and 240 min following an injection of glucose. The measurements of total serum cholesterol and fasting glucose from tail vein blood were carried out using a CardioChek PA system (PTS Diagnostics, Indianapolis, IN, USA) according to the manufacturer’s instructions. Insulin content in the blood was also determined using a Mouse Insulin ELISA kit from ALPCO (Salem, NH, USA) following the manufacturer’s instructions. Insulin content was determined using a standard curve obtained from standards provided by the kit. For the insulin tolerance tests, blood glucose concentrations following an intravenous injection (tail vein) of human recombinant insulin (0.75 U/kg, Lilly) into the mice were measured, which is similar to GTT as outlined above.

### 4.4. Echocardiographic Measurements

Echocardiography of mice was serially conducted using an Acuson Sequoia C512 system equipped with a 15L8 linear array transducer to access left ventricular (LV) function. Briefly, mice were anesthetized with a continuous inhalation of 1–4% isoflurane via nose cone. Mice were placed in the supine position on a warm pad. Hair was removed; pre-warmed ultrasound transmission gel (Aquasonic, Parker Laboratory, Fairfield, NJ, USA) was then applied to the precordial region. The short axis of the left ventricle was selected to capture two-dimensional B-mode images and M-mode at the level of the papillary muscles. Three to six consecutive cardiac cycles were used to assess the M-mode with software. The measurements were performed by an experienced operator in a double-blinded manner.

### 4.5. Histological Analysis

Paraffin-embedded heart tissue slices were fixed with 10% neutral buffered formalin and washed with PBS. For immunofluorescent staining of active caspase-3, fixed sections were incubated with anti-cleaved caspase-3 primary antibodies (1:200 dilution, Abcam cat. no. ab49822) overnight at 4 °C. Following three washes with PBS–Tween each at 5 min, the slides for cleaved caspase-3 staining were incubated with Alexa Fluor 488 goat anti-rabbit IgG (A11001; Invitrogen) secondary antibodies (1:200) for 2 h. Then, the slides were washed (5 × 5 min), and co-staining of DAPI was performed to identify tissue nuclei. Assessment of fluorescent signals was carried out using a Carl Zeiss LSM 700 laser scanning microscope equipped with intuitive ZEN 2009 software. Approximately, five to ten randomly selected fields were used for quantification using NIH ImageJ software. Cardiac interstitial fibrosis was measured and quantified by picrosirius red staining using five to six sections from each heart in chow and HFD groups in both wild-type and PRAK^−/−^ mice. In addition, H&E staining was carried out according to an established laboratory protocol to estimate the myocyte size of each cross section. An approximate 300 myocytes per group obtained mid-distance from the base to apex were randomized to compare myocyte cross-sectional area in each group.

### 4.6. Real-Time Polymerase Chain Reaction (PCR)

Total cardiac RNA was extracted from myocardial tissues in each group with Trizol reagent (Life Technologies, Grand Island, NY, USA). The synthesis of cDNA was performed from 5 μg of RNA. A standing PCR condition was used to amplify the reverse transcribed cDNA (5 μL) at a final reaction volume of 50 μL. Real-time PCR experiments were carried out on a Mastercycler Realplex4 (Eppendorf North America) using a qPCR Kit master mix (Kapa Biosystems, Boston, MA, USA). Primer sequences of ANP, BNP, and β MHC used in these studies were as follows: *Atrial natriuretic peptide* (ANP): forward, CAC AGA TCT GAT GGA TTT CAA GA; reverse, CCT CAT CTT CTA CCG GCA TC. *B-type natriuretic peptide* (BNP): forward, 5-GTC TGG CCG GAC ACT CAG-3; reverse, 5-TGC ACT GGT GTC TTC AAC AAC-3. *Myosin heavy chain beta* (β-MHC): forward, 5-CGC ATC AAG GAG CTC ACC-3; reverse, 5-CTG CAG CCG CAG TAG GTT-3. *Fibronectin Type III Domain Containing 5* (FNDC-5): forward, 5-GAA CAA AGA TGA GGT GAC CA-3; reverse, 5-ACC ACA ACA ATG ATC AGC A-3. VEGF: forward, 5-AGT CCG AAT GCA GAT CCT C-3; reverse, 5-TGC ATT CAC ATT GGC TGT G-3. FNDC-5: forward, 5-GAA CAA AGA TGA GGT GAC CA-3; reverse, 5-ACC ACA ACA ATG ATC AGC A-3. GAPDH was used as the internal control: forward, 5-ACC ACA GTC CAT GCC ATC AC-3; reverse, 5-TCC ACC ACC CTG TTG CTG TA-3. CTG CA GAPDH was used as the internal control.

### 4.7. Terminal Deoxynucleotidyl Transferase-Mediated dUTP Nick-End Labeling Assay

Terminal deoxynucleotidyl transferase nick-end labeling (TUNEL) was performed using a TACS 2-TdT-DAB In Situ Apoptosis Detection Kit (Trevigen, Gaithersburg, MD, USA) according to the manufacturer’s instructions. The number of TUNEL-positive nuclei was counted in each section of the five randomly selected regions; five to six sections were randomized to be used for measurement of positive signals. Three hearts were used for chow and HFD in wild-type and PRAK^−/−^ mice. Approximately 500 cells were measured from each independent group.

### 4.8. Western Blotting

Protein levels were measured by Western blotting using cell lysates (50 μg/lane) as described previously [40]. In brief, the blots were incubated with their respective polyclonal antibodies, which included polyclonal rabbit phosphorylated IRS-1^Ser 1101^, IRS-1 phosphorylated AMPKα, AMPKα, phosphorylated ERK1/2, ERK1/2, and PRAK (Cell Signaling Technology, MA, USA), polyclonal rabbit irisin (Biovision, Milpitas, CA, USA), and polyclonal rabbit β-actin (Cell Signaling Technology, MA, USA) at a diluted concentration of 1:1000. The signals were then visualized by anti-rabbit or anti-mouse horseradish peroxidase-conjugated secondary antibody (1:2000). The results were visualized with Super Signal West Pico ECL chemiluminescence reagent (Thermo-Fisher Scientific). Densitometric analysis for the blots was completed using the NIH ImageJ processing program.

### 4.9. Statistical Analysis

Data are expressed as an average ± SEM of independent experiments. An unpaired, two-tailed Student *t*-test was used to determine differences between two groups. Multiple groups were analyzed using one-way ANOVA followed by Bonferroni post hoc tests. Differences between groups were considered statistically significant when *p* < 0.05.

## 5. Conclusions

This is the first study to demonstrate that knockout of PRAK promotes cardiac dysfunction in response to a high-fat diet, which was associated with the enhancement of cardiac hypertrophy and cardiac remodeling. Furthermore, deletion of PRAK also exacerbated insulin resistance in mice exposed to HFD. The insulin signaling pathway involving phosphorylation of IRS-1, AMPKα, and ERK1/3 was attenuated by deletion of PRAK. Taken together, the studies presented here suggest an important role for PRAK in preserving cardiac function, attenuating myocardial remodeling, and mediating insulin resistance in response to a high-fat diet intervention.

## Data Availability

Not applicable.
